# Beyond modeling abstractions: learning nouns over developmental time in atypical populations and individuals

**DOI:** 10.3389/fpsyg.2013.00871

**Published:** 2013-11-26

**Authors:** Clare E. Sims, Savannah M. Schilling, Eliana Colunga

**Affiliations:** ^1^Department of Psychology and Neuroscience, University of Colorado BoulderBoulder, CO, USA; ^2^Department of Electrical, Computer, and Energy Engineering, University of Colorado BoulderBoulder, CO, USA

**Keywords:** word learning, computational models of development, language development, neural networks (computer), language disorders

## Abstract

Connectionist models that capture developmental change over time have much to offer in the field of language development research. Several models in the literature have made good contact with developmental data, effectively captured behavioral tasks, and accurately represented linguistic input available to young children. However, fewer models of language development have truly captured the process of developmental change over time. In this review paper, we discuss several prominent connectionist models of early word learning, focusing on semantic development, as well as our recent work modeling the emergence of word learning biases in different populations. We also discuss the potential of these kinds of models to capture children’s language development at the individual level. We argue that a modeling approach that truly captures change over time has the potential to inform theory, guide research, and lead to innovations in early language intervention.

## INTRODUCTION

At the core of connectionist models is the idea of modeling change over time. Nowhere is this feature more critical than in the modeling of developmental processes, which by definition occur in time. In this review we focus on the domain of semantic development, specifically early word learning, and highlight the characteristics of the connectionist approach that make it well-suited for modeling developmental processes. We illustrate these characteristics by reviewing several prominent connectionist models of word learning. We argue that, however, most of these models do not fully take advantage of the strengths of connectionist models in capturing the temporality of development. We then turn to our own work modeling developmental trajectories in typically developing children and late talkers. Our approach to modeling word learning has captured intriguing patterns of behavior, produced novel predictions, and has promise for exciting future applications. Throughout the paper, we will explore how computational models of word learning add insight to what is known about this developmental process as well as guide further discoveries.

Connectionist models have made significant contributions to our understanding of various phenomena observed in young children (see Munakata et al., 2008 for a review). In the domain of language development, connectionist models have been used to help explain behavioral data, to test mechanistic accounts of language learning, and to inform big theoretical debates (e.g., Smith et al., 2010; Elman, 2011; Seidenberg and Plaut, in press). In general, connectionist models are well suited to model the time-course and emergent properties of processes. This is because learning in connectionist models is incremental and representations are often under-determined in the beginning and learned as a way to solve a particular task. The current review includes only connectionist models. Connectionist models have the ability to capture processes of change over time as well as to capture multiple timescales of learning, all of which, as we argue in this review, makes them a good candidate model for development. Although connectionism is not necessarily the only way to model these aspects of development (e.g., see Yu et al., 2005; Kemp et al., 2007; Xu and Tenenbaum, 2007; Frank et al., 2009), current research suggests that this is an especially promising approach. We will return to this point in the discussion and touch on other developmental modeling approaches.

To assess the current state of the field, we use four criteria to guide our discussion of prior work (see **Table 1**), and to make the comparisons more informative, we focus on the domain of early word learning rather than attempt to do a comprehensive review of connectionist models of language development. The first three criteria we use have been previously established by Christiansen and Chater (2001), who applied them to a review of psycholinguistic models. These criteria are: data contact, task veridicality, and input representativeness. Data contact refers to how well a model matches empirical data and is able to make novel predictions. Task veridicality involves matching the tasks given to the model to tasks used in the behavioral studies which the model aims to capture. Input representativeness is how well the input to the model captures the input available to the person. In addition to these three criteria, we propose one additional point that is crucial to consider in assessing models of development: temporality. This is a model’s ability to capture continuous change. These four criteria will guide our review of connectionist models of early word learning.

**Table 1:** The four criteria used to assess computational models of early word learning in the current paper.

Criterion	Description
Data contact	The degree to which the model captures the data and makes predictions that can guide and be tested by empirical research
Task veridicality	The match between the task given to the model and the behavioral task used with children
Input representativeness	The match between information given to the model and information available to children in the linguistic environment
Temporality	The ability to capture continuous changes in phenomena

### DATA CONTACT

The first criterion we will apply to models of early word learning is the ability to make contact with empirical data. A good model should accurately capture the phenomenon of interest in order to make meaningful conclusions about what may be driving or supporting that phenomenon. We further propose that making contact with data entails making informative predictions that can guide and be tested in subsequent research. Connectionist models of language development have satisfied this criterion with varying degrees of success.

One prior model of language development that has successfully met this criterion is the word learning model of McMurray et al. (2012). This model learned to map word forms to object referents in an unsupervised learning paradigm. The authors used their model to make contact with a variety of behavioral phenomena. For example, the model showed a pattern of comprehension preceding production of word-referent mappings, a preference for novel referents for novel word forms (consistent with mutual exclusivity), as well as graded object familiarity effects in novel word-referent mapping.

Importantly, McMurray et al. (2012) also demonstrated that their model provided novel insights and predictions. For example, the model was able to effectively learn words even when many object referents were present for a single given word. This suggests that associative learning is sufficient to support learning in highly ambiguous contexts, which young children arguably face when learning new words. The model also showed word learning dynamically unfolding in different ways at different timescales. At a shorter timescale, the model made initial connections between a single word form and a single object referent. At a longer timescale, the model created more efficient and long-lasting representations of word-referent links. From the model, the authors proposed that shorter timescale learning, including processes of word-referent mapping and word recognition, is supported in the moment by competition dynamics. On the other hand, longer timescale learning, the retention and refinement of initial mappings, is driven by slower associative learning dynamics.

Li et al. (2004) also made contact with data in their model of semantics and phonology in lexical development. In this model, phonological word form and semantic word meaning representations were formed initially, and were then organized and linked together through associative learning. Among other results, this model captured age of acquisition effects in word learning, showing that learning time was positively correlated with vocabulary size once the lexicon had reached a certain size. In terms of insights and predictions, the authors used their model to show that lexical category representations need not be innate. Mappings between phonological and semantic categories can be learned given the kind of input that is available in the linguistic environment of young children.

Finally, Yu (2005) presented a model of how category learning may interact with word learning early in development. This model was set up to explore a proposed feedback loop between perceptual features of objects and linguistic labels in children’s category learning. Although Yu accurately captured the reinforcing relationship between category and language learning in children, the model did not clearly demonstrate the dynamics of the bidirectional relationship in question. The model results demonstrated that learning was improved by the presence of word representations compared to when they were removed, though further testing would be needed to strengthen the claim of bidirectionality. In particular, this model would benefit from tests of interactions over time, a point we will return to later when we discuss the fourth criterion of models of language development.

### TASK VERIDICALITY

The next criterion we will explore with respect to connectionist models of semantic development is the match between the task given to the model and the behavioral task used with people, and in this case, children. The need for a model to capture realistic components of experimental tasks must also be balanced with the need to isolate specific processes that may be at work. That is, modelers must decide which aspects of a given task must be included in a model and which are superfluous in terms of explaining phenomena. Although the ultimate goal would be to construct a model that could capture many different tasks, along the lines of constructing a unified theory, adding complexity does not always make for better explanatory value. For example, a hypothetical model that captures visual, auditory, and semantic processing in children’s word learning may reproduce behavior more completely, but may not give much insight into each specific process. Various models of language development have struck this balance in different ways.

Regier’s (2005) model achieved veridicality in both the training and testing tasks implemented in the model. In this model of word learning, word forms and word meanings were presented and organized into clusters of exemplars, and associative links between these clusters were learned. Over time, the dynamics of the network adjusted the weightings of various dimensions of form and meaning, simulating the dynamics of selective attention to features in word learning. The training task in this model, in which word forms and meanings were presented simultaneously, captured the typical situation of a child receiving simultaneous visual and label input as their parent teaches them new words. To test the model, Regier simulated a typical forced choice word learning task. After exposure to a novel word pattern, the model was presented with the target word form and had to correctly activate the target meaning from among multiple distractor patterns. This simulation is a good match to a common behavioral task administered to children.

Another example of task veridicality can be seen in Mayor and Plunkett’s (2010) model of word learning. This model did a particularly good job of isolating specific processes that seem to be especially important in word learning. In an early stage of learning, the model was presented with visual object and acoustic language input, and each type of input was processed separately. Each type of input became organized into similarity-based categories, simulating a child learning perceptual patterns in the environment in an unsupervised manner, without explicit teaching signals or feedback. In a subsequent stage of learning, visual, and auditory input were presented simultaneously and became linked through associative learning, simulating supervised learning of word-object pairs. In this way this model set out to test the idea that specific, distinct learning processes drive language development at different times.

Li et al. (2004) achieved good task veridicality in the training scheme for their model. Phonological word form and semantic word meaning representations were presented simultaneously and interacted bidirectionally over learning. However, the veridicality of the testing tasks used in this model is not as clear. For example, in a test of comprehension the model was first given a phonological word form representation to process, which then fed forward to semantic processing, and finally produced a word meaning. The model was tested for production in a similar way, beginning with word meaning inputs. It is questionable whether performance on real comprehension and production tasks proceeds in this feed forward fashion. A more realistic task may instead include bidirectional interactions at the time of testing as well as training, with partial activations of word forms and meanings mutually influencing each other.

Overall, several models of early word learning have shown strong task veridicality, helping them in turn make contact with behavioral data. Yet another important component of such models that goes hand in hand with incorporating realistic training and testing tasks is using plausible input patterns. That is, a well-designed task simulation is no longer as realistic and meaningful if the input to that task differs dramatically from information that is actually available to young children learning language. Therefore, the need for input that accurately captures realistic and important information available in a child’s linguistic environment is the next criterion we will turn to.

### INPUT REPRESENTATIVENESS

Christiansen and Chater (2001) defined input representativeness as the match between information given to the model and information available to the person. In the case of models of semantic development, this means designing inputs for the model that capture realistic patterns of information that are available in the linguistic environments of young children. Like the previous criterion discussed, input representativeness is also related to the idea of isolating specific processes using a model. To guide the design of input that is both simplified and representative, it is helpful to focus on the information that is most relevant to a process and to exclude irrelevant information. For example, in a model of visual processing, it would be important to capture information such as form, orientation, lighting, and contrast. However, while ultra violet light is technically a piece of information present in the system, it is not relevant to human visual processing and therefore would be irrelevant information for such a model. In this same way, it is important in models of language development to determine what information, such as semantic, perceptual, social, or phonological information, is relevant input to the particular phenomenon of interest.

One example of good input representativeness can be seen in Yu’s (2005) model of word and category learning. To create input for the model, Yu collected visual and acoustic data from adult subjects. Multiple subjects were recorded while reading a storybook as if they were narrating to a young child. This method captured realistic co-occurrences between the visual objects that were seen on a page and information that was narrated in speech. Importantly, this input captured not only real information in an environment that would be experienced by a young child, but also the temporal order of this information. The combination of visual and acoustic information yielded model input that was highly representative of information available to young children learning language.

As discussed earlier, Mayor and Plunkett (2010) presented a model that learned word-object associations through an unsupervised followed by a supervised phase of learning. The authors designed input patterns that represented the kinds of information that young children would actually get in these two kinds of learning contexts. Initially, during unsupervised learning, the model was given uncorrelated visual object and acoustic word token representations. Later, during supervised learning, the model was given more structured input with simultaneous presentations of a word with its corresponding object representation. The authors referred to this second stage as joint attention, further showing the link between the input and the specific task that was simulated at that point in the model. In this case, the authors achieved input representativeness by matching the characteristics of the input to the specific learning task that was implemented at a given point in time.

Finally, another model discussed earlier demonstrates the balance between input representativeness and isolating specific processes. In their model of word learning, McMurray et al. (2012) designed the input such that auditory word forms and visual object categories were represented locally, by single units in the network. Learned associations between these units were represented in a hidden layer of lexical units. The hidden layer contained many more lexical units than either the word or category layers in order to better capture learning. Altogether, this input was somewhat removed from the level of information that would be readily available in the environment of a young child. The authors’ use of localist representations does not allow them to capture certain finer details that real children use in word learning, such as visual scene variations and similarities that support object categorization. However, the authors chose to use localist rather than distributed representations because they offered certain advantages. This input allows the authors to isolate specific learning mechanisms, such as competition between potential lexical representations in referent selection. As the authors discussed, their simplifications in the model helped strengthen their theoretical point about learning mechanisms that may be crucial in early language development.

Of note, both the criteria of input representativeness and task veridicality are important for using a model to identify meaningful theoretical implications. The tasks that are simulated and the input presented to a model must represent at least some characteristics of the tasks and information encountered by children learning language. At the same time, both of these factors must be balanced with the isolation of specific processes. Isolating processes that are theorized to be key to language development allows researchers to conduct targeted tests of theory within their models. Models of language development must strike this balance between accurately representing the context of learning while targeting specific variables and processes that underlie and support the specific developmental phenomena of interest. We now turn to a final proposed criterion for evaluating connectionist models of development.

### TEMPORALITY

The criteria discussed so far are important to consider for any connectionist model. We propose a final criterion that sets developmental models apart: temporality, or the ability to capture continuous changes and the processes that drive that change. Rather than modeling discrete developmental stages, models that account for temporality capture an ongoing process of change. These changes can be characterized as occurring over time, but also could be, more generally, the sequence of developmental milestones reached, or any other continuous, sequential measure. The key is that the processes of change posited by the model can drive change through the appropriate series of milestones. Connectionist models are particularly well-suited to incorporate temporality and have been used to explore learning over multiple timescales. For example, such models can be used to investigate the formation of connections over time as they emerge and develop. However, many models in the domain of early word learning have not fully captured development as a continuous process. Here we will evaluate the connectionist models discussed above with respect to the final criterion of temporality.

First, although Yu (2005) modeled word learning, the model was not evaluated in a way that measured changes over time. The model results only represented the end point of learning in different conditions. Although Yu used the model to explore the idea of a developmental feedback loop between word and object category learning, the dynamics of this relationship were not explored over time. This model did capture behavioral results observed in young children’s word learning, but as presented, it did not demonstrate how word learning unfolds as a developmental process.

The other connectionist models discussed above captured developmental processes of language learning more directly by modeling specific changes that take place over multiple time points of learning. However, in two of these models the developmental change was built into the model a priori. For example, in one model there was a major developmental change built into the input (Mayor and Plunkett, 2010). As discussed earlier, Mayor and Plunkett implemented two stages in their model of early lexical learning. An early, unsupervised learning stage was meant to capture the emergence and refinement of perceptual categories in infancy, and a subsequent supervised learning stage was meant to capture word learning through joint attentional events. Although these stages are theoretically grounded and development within each stage was explored, the process of transitioning between these two stages was not captured by the model. Instead, a qualitative change in word learning was represented by an abrupt change in input and training regime, which likely happens as more of a gradual transition in real children.

Another model that does not fully meet the criterion of temporality is that of Li et al. (2004). In this model, the authors also posited two stages of learning: an initial stage in which learning helps establish a rough topography of lexical categories in similarity space and another stage in which learning fine-tunes these representations. The change from one stage to the next was modeled as a gradual transition that unfolded over time, however the parameters guiding this transition were specified a priori in the model. Similarly to Mayor and Plunkett (2010), Li et al. (2004) did investigate continuous developmental changes taking place across stages and throughout the transition period. However, the developmental transition between those stages did not emerge from the modeled processes alone. Therefore, this model captured some extent of temporality, but ultimately resorted to an a priori change in parameters to capture an important part of the developmental process.

The final two models that we have focused on thus far more fully meet the criterion of temporality. These models captured continuous change over time through emergent dynamics rather than changes to input or parameters during the course of learning. For example, Regier (2005) actually made a theoretical point of using a single mechanism to model several word learning phenomena. Some researchers have posited a mechanistic shift from associative to referential learning to explain developmental changes in behavioral patterns of word learning. Regier’s model demonstrated that behavioral patterns previously considered evidence for this shift can actually be explained by the dynamics of a single mechanism over time.

Another example of good temporality in a model can be seen in that of McMurray et al. (2012). In their model of word learning, the authors captured continuous developmental change over two time scales. Importantly, the mechanisms at work at each time scale emerged from the network rather than being implemented through explicit changes in the input or architecture. This model showed that immediate, short-term, “situation time” learning was driven by competition dynamics whereas slower, long-term learning and retention were driven by associative dynamics. These dynamics were continuously at play and interacted with each other over development in the model, resulting in temporality.

Taking this kind of developmental perspective in modeling, that is, striving to meet the criterion of temporality, could have important implications and applications. For example, capturing change over time could help to leverage the information we have about children at one point in time to predict how they will learn at a later time point and their future outcomes. This approach could perhaps even provide new opportunities to intervene and improve the learning process for children. In current, ongoing work in our lab we aim to do just this with a developmental model of word learning.

### OUR APPROACH

Our work builds on prior connectionist models of language development. We are interested in exploring how skilled word learning continuously develops and may emerge from general domain processes. This perspective is in line with other connectionist work that demonstrates how complex, smart behavior can emerge from simple learning rules acting over distributed representations (e.g., Rogers and McClelland, 2006; Elman, 2011; McMurray et al., 2012).

**FIGURE 1:**
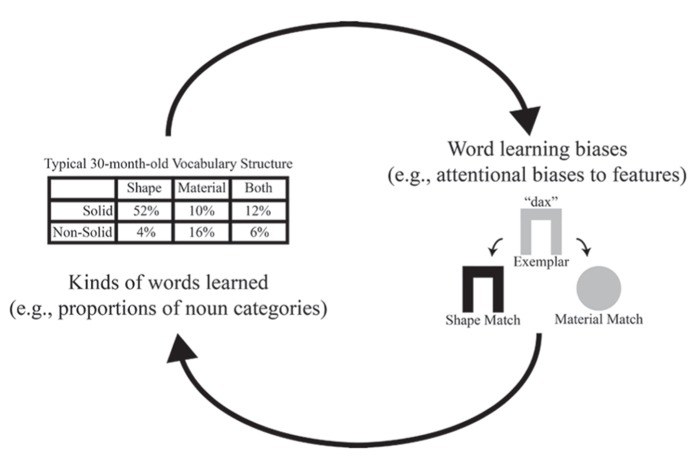
**Schematic of the hypothesized developmental feedback loop.** On the left side are the kinds of words that children learn (represented here as proportions of different categories of nouns, as we used in our model). On the right side are attentional biases to features in word learning (represented here with a classic novel noun generalization task).

The phenomenon of interest is this: children become skilled learners, at least in part, because they know about the different kinds of properties that are relevant for categorizing different kinds of things. Typically developing children show *word learning biases*: they generalize names for solid objects by shape and names for non-solid substances by material (e.g., Landau et al., 1988; Jones et al., 1991; Soja et al., 1991; Soja, 1992; Samuelson and Smith, 1999; Colunga and Smith, 2008). These are termed the shape and material bias, respectively. The evidence suggests that children learn how to learn nouns – and specifically learn how different kinds of properties are relevant for different kinds of things – as a consequence of learning names for things. Each noun the child learns appears to teach the child something general about how to learn new nouns that name things of that same kind, and critically, at the same time, this learned general knowledge constrains and facilitates the types of nouns the child will learn next. This self-constructing developmental loop involving word learning and category learning (see **Figure 1**) has been partially implemented as a connectionist model. A simple neural network trained using contrastive Hebbian learning on a vocabulary structured like that of the average 2-year-old will show attentional biases akin to those of the average 2-year-old when learning new words (Colunga and Smith, 2005).

This relationship between vocabulary structure and word learning biases has been typically characterized in one of two ways: abstract knowledge guides, facilitates, and indeed allows word learning, or the words that have been learned give rise to, create, and in fact constitute generalized knowledge about word learning. Connectionist models implement a version of the latter account – without being given abstract, or rule-like knowledge, the networks acquire different biases for solids and non-solids as they learn individual categories of solids and non-solids instance by instance. Importantly, this modeling approach gives the power to test proposed causal accounts of word learning biases, a point we will return to when we discuss recent results from our lab.

The work reviewed here extends these previous findings, and speaks to the criterion of temporality, in two important ways. First, we look at the relationship between vocabulary structure and word learning biases not only *after* the vocabulary of the average 2-year-old has been learned, but *while* this vocabulary is acquired. Second, we look at the relationship between vocabulary structure and word learning biases for children who are not average, but rather late or early talkers relative to their peers. Finally, we look at this relationship based on the vocabulary structure and word learning behavior of individual children between the ages of 18 and 30 months of age.

In the remainder of this paper we first review the evidence for this interactive link between vocabulary growth and the emergence of word learning biases. Then we will introduce our modeling approach and review some results of this approach, both from our neural network model and corresponding behavioral studies of young children. Finally, we will discuss implications and future directions for this developmental approach to modeling word learning.

## WORD LEARNING BIASES

Although there is some debate over the origin of word learning biases (e.g., see Samuelson and Bloom, 2008) some researchers have linked their emergence to the developmental process of vocabulary acquisition. By looking at the shape bias over time, research shows a larger developmental story involving an interplay between attentional biases and vocabulary learning. One study on the emergence of the shape bias tested children longitudinally on their attention to shape in generalizing a novel label (Gershkoff-Stowe and Smith, 2004). These researchers also collected diaries from parents tracking children’s vocabulary growth. The results showed that children’s attention to shape increased concurrently with increases in the number of nouns in their vocabularies. This suggests that the shape bias emerges in part due to the process of vocabulary growth itself. Another study provides evidence that the emergence of the shape bias can also influence subsequent vocabulary growth. Smith et al. (2002) intensively trained 17-month-old children on labels for novel shape-based categories of objects. The children exposed to this training not only developed a shape bias earlier than is typically seen, they also showed a dramatic increase in vocabulary size over the course of the study compared to a control group. These results suggest that the development of the shape bias accelerates children’s learning of object names outside of the lab. Together these studies suggest that (1) the shape bias emerges out of language development, specifically word learning, and (2) as the shape bias emerges it can in turn exert an influence on further vocabulary growth.

Connectionist models of word learning have also helped contribute to understanding of how children may acquire attentional biases in noun learning. For example, Colunga and Smith (2005) trained a connectionist model on input patterns structured like a typical early child noun vocabulary. These input patterns represented solid objects and non-solid substances which varied systematically in the key features of shape and material. The network input was designed to capture the correlations between solidity and feature for different types of words observed in children’s early noun vocabularies (Samuelson and Smith, 1999). Colunga and Smith (2005) then tested the network for generalization to novel test patterns, and found output patterns consistent with shape and material biases. That is, in the generalization test the networks represented novel solid patterns based on similarity in shape rather than material, and represented novel non-solid patterns based on similarity in material rather than shape. This work shows that given input with the correlational patterns found in a typical early child noun vocabulary, a neural network model can similarly acquire selective attentional biases like those observed in toddlers.

In sum, prior research on the origins of the shape bias in toddlers suggests that attentional biases and vocabulary acquisition interact and build on each other over time. That is, selective attention and word learning are both key components of a self-constructing developmental feedback loop in children’s early noun learning. Connectionist models of word learning may be a particularly useful way to further investigate and guide empirical studies of this loop. For example, the model of Colunga and Smith (2005) captured part of the feedback loop, showing that the typical early child vocabulary composition has a structure that is sufficient to support the development of attentional biases in generalization. In more recent work in our lab, we have used this modeling approach to further explore the developmental feedback loop in noun learning in a few different ways.

## MODELING THE EMERGENCE OF BIASES

In our work, we use a connectionist model that simulates how children learn words via selective attention to object features, or biases. We focus on a developmental feedback loop in word learning between the kinds of words a child knows and how they learn new words. Importantly, we implement this is in a temporal way, by stopping the network at multiple points during training and testing its performance to capture the trajectory of bias emergence and interactions within the loop. This methodology speaks to our fourth criterion of a good developmental model of language acquisition. In this way, we aim to capture the interactions between different kinds of attentional biases and different types of words which could occur in children’s learning as their vocabularies grow. Indeed we have tested predictions made by the networks in a longitudinal study of 18- to 30-month-old children.

We use our model primarily to address the point of temporality, investigating the emergence of word learning biases as vocabulary grows over time. But how does our model measure up against the other key criteria of models of language development? As we will discuss shortly, our model is low in input representativeness. In order to focus on specific processes in learning we must greatly reduce the level of detail of the linguistic information that real children encounter. We do this in principled ways that we believe help us get at our key theoretical questions. By including minimal information in our input patterns, we are able to eliminate other possible factors which could affect word learning and focus specifically on the effect of vocabulary structure on word learning. However, the input to our model represents a subset of the words that a typical child is expected to learn within the first few years of life; therefore, this does not represent all of the linguistic input that children are truly exposed to, as children hear more words than they learn. In terms of task veridicality, we strive to meet this criterion by implementing a simulated version of a common word learning task that is given to children. Finally, we believe our model makes good contact with the behavioral data, as we will discuss in reviewing results from our lab.

**FIGURE 2:**
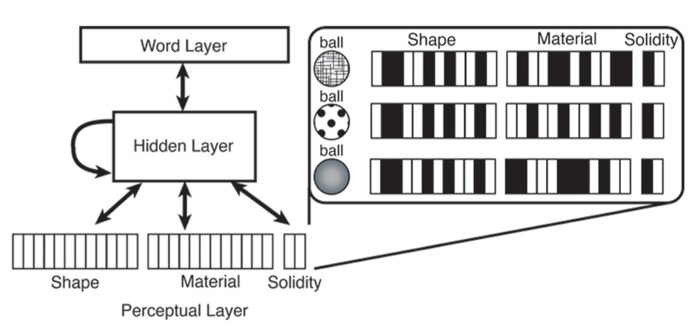
**Network architecture of our word learning model and example input patterns for a solid shape-based noun category**.

Our model is a neural network implemented in the software package Emergent (O’Reilly et al., 2012). It uses the Leabra algorithm (Local, Error-driven and Associative, Biologically Realistic Algorithm), which combines both Hebbian and error-driven learning. The network architecture is adapted from Colunga and Smith (2005) and is shown in **Figure 2**. The word layer represents word labels in a localist way. Previously, we discussed another developmental word learning models’ use of localist rather than distributed representations of labels with respect to our third criterion: input representativeness. A distributed pattern of representation provides a model with more information about a given word and how it is similar to other words. This kind of information could be phonological or semantic properties, for example, which are arguably useful in word learning. However, in our model we argue that this kind of information is not necessary to form attentional biases and we focus relevant input to only certain perceptual features. As seen in **Figure 2**, the only distributed patterns of representation in our model are those of the perceptual features, the shape and material, of an item. Solidity is represented discretely, with one unit representing solid and one unit representing non-solid. All of these layers are connected together by a hidden layer which allows the network to form associations between the different perceptual features of the word and the word label itself.

**FIGURE 3:**
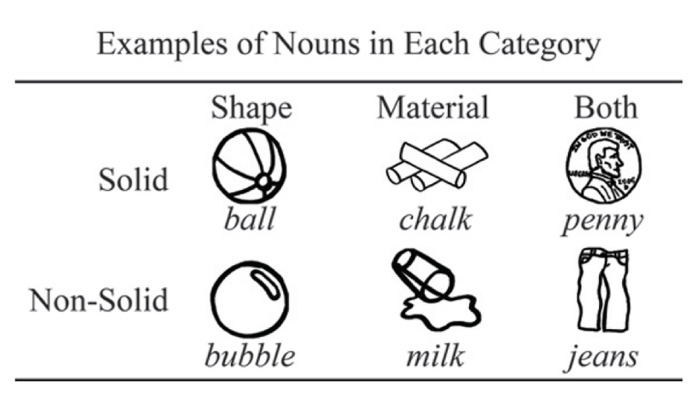
**Noun categories based on adult judgments of solidity (solid or non-solid) and characteristic feature (shape, material, or both) for nouns in the MCDI vocabulary checklist.** Examples of words in the MCDI that fit into each of the six categories of interest are shown.

The network is trained with input structured like the noun vocabulary of a typical 30-month-old child. This input structure represents the endpoint of a learning process that we observe over time in the model. This is analogous to a longitudinal study of vocabulary growth in children which ends at 30 months of age. The main difference is that in our model we must specify the range of words that are to be learned over time, whereas in children this learning takes place naturalistically within the linguistic environment. To capture this typical early vocabulary structure, we used the 30-month-old vocabulary norms of the MacArthur-Bates Communicative Development Inventory (MCDI; Fenson et al., 1993). We divided this vocabulary into six categories based on solidity (solid or non-solid) and characteristic feature (shape, material, or both); see example words in each category in **Figure 3**. These category divisions were adapted from those used in Colunga and Smith (2005) and were based on adult judgments of solidity and characteristic feature for words in the MCDI ^1^. These categories were then transformed into percentages, as shown in **Table 2**. These percentages represent the typical structure of an early child vocabulary, and can be used to create an input vocabulary for the model; in our model we created a 100 word vocabulary input containing the six categories of interest in the proportions shown in **Table 2**.

**Table 2:** Noun categories based on adult judgments of solidity (solid or non-solid) and characteristic feature (shape, material, or both) for nouns in the MCDI vocabulary checklist.

	Shape	Material	Both
Solid	52%	10%	12%
Non-solid	4%	16%	6%

*Percentages indicate category representation for a typical 30-month-old vocabulary. An example noun from each category is also provided.*

During training, a word, such as *ball*, is paired with a pattern of features across the perceptual layer (see sample input patterns in **Figure 2**). To simulate learning words for categories of items, each word is presented multiple times and feature patterns along the perceptual layer are manipulated in specific ways. *Ball*, for example, is a word for a solid item characterized by shape; therefore each instance of the word *ball* is represented as the same shape but can vary in material. To implement this computationally, each time the network sees the word *ball*, the pattern along the shape layer (representing, e.g., a round shape) remains the same, but the pattern along the material layer is randomly varied. This is done for each of 100 words in the typical 30-month-old vocabulary structure input presented at each epoch.

In order to capture the developmental trajectory of word learning in the model, we stopped the network at multiple points during word learning and measured its performance on a virtual novel noun generalization (NNG) task. The network was tested after it had learned a certain number of words: at 5 words learned, 10 words learned, and so on. This is a vital feature for a suitable developmental model. By tracking the progress of learning at different time points, based on amount of words learned, we can analyze the emergence and development of word learning biases. The key component which helps our model meet the criterion of temporality is that we not only track development in a temporal manner, but we model it without changing any network parameters. Rather than trying to represent development as discrete stages, we model it as a continuous process and thus focus on the emergence of word learning biases resulting solely from the structure present in the vocabulary input.

Testing was implemented by simultaneously presenting the network with a triad of novel patterns: one exemplar pattern, one pattern matching the exemplar in shape, and one pattern matching the exemplar in material. This virtual NNG task was implemented with both solid and non-solid patterns in order to see whether the network preferred shape or material in the context of each kind of item. In this way, for both the training and testing of the network, we attempt to achieve meaningful task veridicality. Training of the network is similar to a child’s word learning in the real world: they are presented with objects (perceptual features) and corresponding labels multiple times as they learn new words. For testing, the task we have implemented is directly analogous to a forced choice NNG task, as the network is presented with an exemplar, and then has to determine which of the two different kinds of feature matches is most similar to the exemplar. Both of these tasks are representative of the behavioral tasks which we aim to model, therefore we achieved good task veridicality in our model.

The network’s feature preference (i.e., its attentional bias) was measured based on similarities of hidden layer activations between the exemplar and the two matches. If the hidden layer activations of the exemplar and the shape match were more similar than those of the exemplar and material match, then the network was considered to have a shape bias. If the reverse was true, then the network had a material bias. In this way, we obtained a measure of the extent of attention to each feature in the network over time and we were able to pinpoint the particular point of bias emergence throughout the course of word learning. Using this approach, we have recently explored how different feature biases emerge and develop.

In Schilling et al. (2012), we used this method to study the interactions between two different kinds of biases, the shape bias and the material bias. We ran 10 instances of the network as described above, then identified the point in the course of word learning where the shape bias emerged for each individual network. We found that, as the shape bias emerged for solid items, the network’s attention to material for non-solid items diminished. This finding predicts that children must focus their attention on certain features, such as shape, when developing ways to learn new words and concurrently pay less attention to other features, such as material.

One of our criteria for a developmental language acquisition model was data contact. The results for Schilling et al. (2012) make an important prediction about how children shift and focus their attention to object features in learning new words and we can test this prediction in real children. In Sims et al. (2012), we did this in a longitudinal study of 18- to 30-month-old children. We recruited 20 participants for a monthly, yearlong study beginning at 18 months old age. At each visit, each child was administered a NNG task for both solid objects and non-solid substances to measure their extent of attention of object features and thus their bias development. We also measured their vocabulary growth with the parent-completed MCDI vocabulary checklist. We found that the network predictions were confirmed in children; as children’s attention to shape on solid NNG tasks increased around the emergence of the shape bias, their attention to material on non-solid tasks decreased. As a model of word learning bias development, our model satisfies the criterion of data contact and provides novel, meaningful predictions about child language learning.

The aforementioned work makes useful conclusions about one side of the developmental feedback loop, attentional word learning biases, but what about the other side of the loop: vocabulary development? Could there be meaningful interactions in the kinds of words a child learns around the pivotal point of the emergence of the shape bias? These are questions which we hope to address in future research. Recently, in Sims et al. (2013), we have begun to use our model to investigate changes in vocabulary structure around the emergence of the shape bias for solid objects. This work predicts that as attention to shape increases for solid objects, the number of shape-based words which the network learns increases and at a relatively faster rate than that of the material-based words. These results hint at the possibility that certain types of words are learned better or worse at specific moments in development depending on how attention is deployed to specific object features. The state of the vocabulary structure analysis in children is currently inconclusive, but it is the topic of ongoing research in our lab.

From this work, we see that there may be interactions between both shifts in attention and the kinds of words that a child learns. Our neural network model is an important tool for data analysis because it allows us to make predictions about empirical data and to guide behavioral data analysis. Additionally, our model gives us some insight into the potential mechanisms of bias emergence. The model is given only the input of the structure of a child’s vocabulary sans any phonological or semantic information and it learns word learning biases just as a child would. This is important because it supports the notion that word learning biases need not be an innate mechanism, but rather a phenomenon which emerges from the structure of a child’s noun learning environment. The combination of our model and behavioral data provides useful insight into the developmental trajectory of word learning in toddlers.

## MODELING DIFFERENT POPULATIONS OF CHILDREN

So far we’ve reviewed how our connectionist network can capture the developmental trajectory of vocabulary growth and the emergence of word learning biases. The next question is, can we use this method to model different kinds of developmental trajectories? This approach may be useful for capturing and explaining meaningful differences among populations of children. Of specific interest are children who fall at two ends of a language endowment spectrum: late and early talkers. These are children who score on the lower and upper ends, respectively, of normative language production measures. These two groups of children differ significantly; a 2-year-old in the bottom 10th percentile may produce around 10 words whereas a 2-year-old in the top 10th percentile will produce well over 300 (Fenson et al., 1993). Late talkers in particular are children who are delayed in vocabulary development, but otherwise show no cognitive or neurological deficits. While some of these children catch up in vocabulary development, others are later diagnosed with Specific Language Impairment, and vocabulary measure norms are not sufficient to predict which children will catch up and which will lag behind (Thal et al., 1997; Rescorla, 2002; Desmarais et al., 2008). It is not yet clear why late and early talkers differ so much in language production, nor why individual late talkers can have such varied outcomes. It may be the case that these populations of children can be characterized by different approaches to word learning, a possibility that we explore with our model.

Variations from the typical trajectory of language development may be due to an interruption in the developmental feedback loop. Referring back to **Figure 1**, we see that the developmental feedback loop demonstrates a relationship between vocabulary structure and word learning biases, so a disruption in either of these factors can cascade and affect word learning. For example, late talkers have relatively small vocabularies and therefore may have less varied and potentially atypical vocabulary structures. Because of this, late talkers can miss out on useful correlational patterns present in the structure of larger, more typical early child vocabularies. For example, say a late talker knows just 13 words, as shown in **Table 3**. This hypothetical late talker knows 10 solid words, four of which are based on shape, another three based on material, and the last three characterized by both shape and material. With this information, there is not enough of a difference in frequency in the kinds of words this child has been exposed to. This child lacks information which typically developing children have (a vocabulary in which most solid words are characterized by shape) thus this late talker has no basis to support the development of a shape bias for solids (or any other bias for that matter). This shows that late talkers can have a deficit in one piece of the loop, vocabulary structure. Subsequently, this gives children who are late talkers less of a basis on which to build on the other piece of the loop, developing helpful word learning biases. In this case, it is the left side of the developmental feedback loop, the vocabulary acquisition, that is disrupted. Alternatively, the problem could be in the arrow from vocabulary structure to word learning biases; late talkers may struggle with leveraging the correlational structure in the words they already know to abstract higher level attentional biases. We investigated these possibilities in studies of early talker and late talker toddlers and networks.

**Table 3:** Example of possible vocabulary proportions for a late talker toddler.

Example late talker vocab (no. of words)			
	Shape	Material	Both
Solid	4	3	3
Non-solid	0	0	3

*For solid words, this child knows approximately equal proportions of words characterized by shape, material, and both shape and material. If a child were developing a bias for solid words based on this vocabulary structure, it would be difficult for them to discern what is an important feature for identifying novel solid words.
*

**FIGURE 4:**
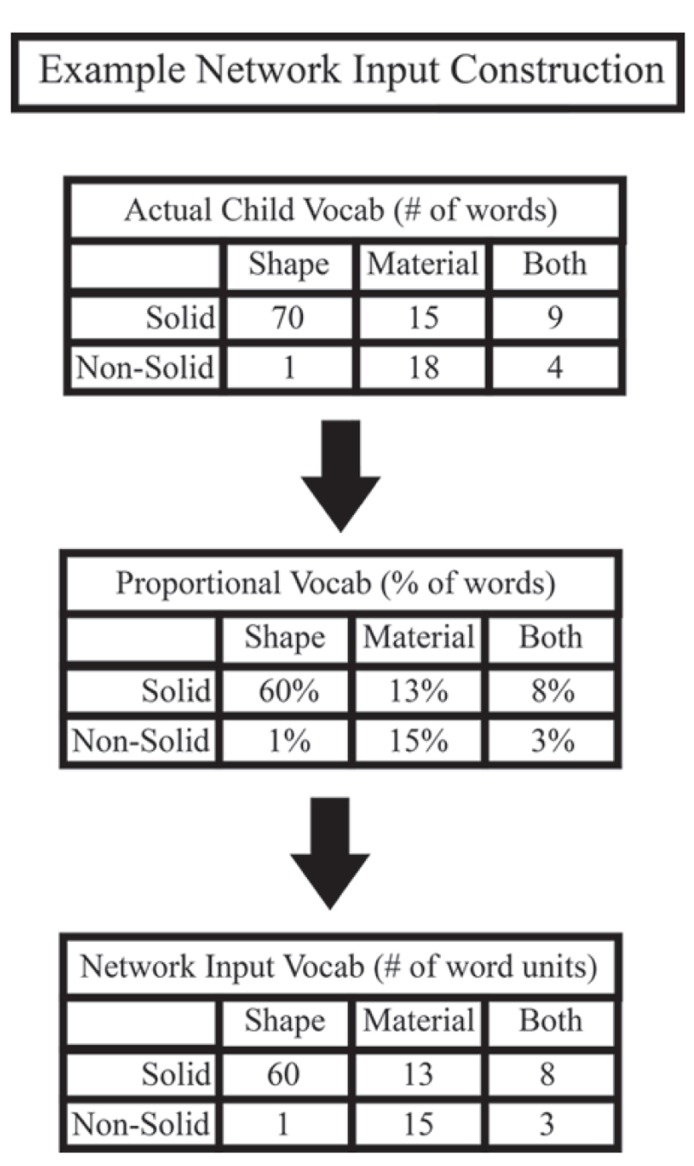
**Example of how our network input is constructed from children’s vocabulary data.** First, the nouns that children know from the MCDI are broken into the six categories of interest. These raw numbers are then turned into percentages, normalized by the total number of nouns a child knows. This intermediate representation best illustrates what we mean by vocabulary structure. Finally, these percentages are used to create 100 noun representations that are used as training input for the model.

In one study from our lab, we compared the vocabulary structures of early and late talker children (Colunga and Sims, 2011). We examined age-matched groups of early talker (above the 75th percentile on the MCDI) and late talker (below the 25th percentile) 18- to 30-month-old toddlers. Children’s vocabulary structures were analyzed by sorting known nouns into the six categories described earlier, with solidity (solid object or non-solid substance) crossed with characteristic feature (shape-based, material-based, or both shape- and material-based). **Figure 4** shows an example of how raw vocabulary, the words a child knows, is used to create network input patterns. Once the words in the child’s vocabulary are grouped into the six categories of interest, the vocabulary is then re-represented as word type proportions with respect to the total number of words in the vocabulary. These proportions are then scaled to 100 word units to create the network input patterns. In this way, it does not matter if the child is an early talker and knows 200 words or a late talker and knows just 10 words. Our model’s input patterns are proportions of kinds of words and therefore capture the structure present in children’s vocabularies while controlling for vocabulary size differences. Although both late talkers and early talkers knew more words for solid objects characterized by shape than any other category, the vocabularies of these two groups differed qualitatively. The most striking difference was seen in the variability among each group; late talkers showed greater variability in their vocabulary structures compared to early talkers. While early talkers’ vocabularies tended to have the same structure as that of the 30-month MCDI norms, late talkers’ vocabulary structures took on different forms.

We next used network simulations to explore possible ramifications of these different vocabulary structures (Colunga and Sims, 2011). Each individual early and late talker child’s vocabulary structure was given as input to our word learning network described earlier. The networks trained on early talker vocabularies all developed a shape bias for solids, and the majority also developed a material bias for non-solids. That is, these networks correctly extracted the kinds of attentional biases that have been shown to be helpful in young children’s word learning. On the other hand, most but not all of the networks trained on late talker vocabularies developed a shape bias for solids, very few developed a material bias for non-solids, and several actually showed an overgeneralized shape bias for non-solids. The predicted generalization patterns for networks trained on late talker vocabularies significantly differed from those of networks trained on early talker vocabularies.

This first exploration of the developmental feedback loop in different populations of children showed that these children do indeed know qualitatively different kinds of words. Further, the network simulations suggested that these vocabulary differences may carry through and impact the kinds of word learning biases that different groups of children develop. But are differences in vocabulary structure linked to qualitative differences in word learning biases in real children? To answer this question, we brought a sample of early and late talker 18- to 22-month-old toddlers to the lab to test the predictions of our network (Colunga and Sims, 2012). We tested children on two versions of the NNG task, one involving solid objects and one involving non-solid substances. Early talker toddlers showed a robust shape bias for solids as well as a robust material bias for non-solids. Late talkers also showed a robust shape bias for solids. While late talkers as a group did not show any consistent bias for non-solids, four out of nine of the children in this group showed an overgeneralized shape bias for non-solids, as predicted by the network simulations.

These results provide further evidence for the link between vocabulary composition and word learning biases, that is, the developmental feedback loop. This link has previously been suggested and supported by other research, but the work from our lab makes some new contributions. First, this work uses the powerful approach of modeling language development to make predictions and guide analysis of behavioral data. We use this approach in a novel way, helping to fill in the developmental picture of the relationship between vocabulary structure, that is, the kinds of words that children know, and attentional biases in different populations of children. This approach helps us to isolate the specific role of vocabulary structure and explore how differences in it can result in different attentional biases in early and late talkers. As confirmed by behavioral data, our model showed that early talkers develop helpful word learning biases earlier than the typical population of children, and that late talkers actually do exhibit an early (and sometimes overgeneralized) shape bias.

Second, this work and our modeling approach have provided insight into possible mechanisms that may be driving differences in ability along the language endowment spectrum. Our model and behavioral studies show that early and late talkers exhibit intriguing patterns of differences in both the vocabulary composition and attentional bias components of the developmental feedback loop. By isolating these processes and focusing on a specific piece of children’s linguistic environments, the model results suggest that both parts of the self-constructing loop are disrupted in late talkers relative to early talkers and typically developing children.

It is important to note that, thus far, our work in modeling different populations of children has not incorporated temporal analysis. The aforementioned work in both networks and children has focused on the presence or absence of word learning biases at one point in time rather than interactions in bias emergence which occur as attention shifts throughout the trajectory of word learning. In future work, we plan to incorporate temporality into our models of late and early talkers. It is possible that different groups of children exhibit different kinds of interactions of word learning biases and vocabulary structure which could be predictive of future outcome. Exploring how the trajectories of learning differ for children at different points along the language endowment spectrum has the potential to guide diagnosis and intervention. Identifying differences in word learning interactions in early and late talkers could lead to intervention techniques and even early diagnosis of persistent late talkers (i.e., children with Specific Language Impairment). In the next section we will further discuss potential extensions and applications of this work.

## WHAT’S NEXT?

The use of computational models has deepened our understanding of the processes that drive language development. Our work reviewed here shows that a simple connectionist network, embedded in a structured environment, can capture critical characteristics of the developmental trajectories of the emergence of word learning biases in typically developing children as well as in late talkers. One direction that we are pursuing with our model is further exploring the full developmental feedback loop between vocabulary growth and selective attentional biases. So far we have good evidence, both from our model and longitudinal behavioral data, for the emergence of and interactions between the shape and material biases in early word learning. This supports one part of the developmental loop: as vocabulary structure emerges, the development of attentional biases is supported and unfolds dynamically over time. That is, our work both supports and establishes a detailed developmental picture of how word learning leads to the emergence of attentional biases to object features.

Yet this proposed developmental loop is also characterized by a complementary process through which attentional biases guide and influence the course of subsequent vocabulary growth. As discussed earlier, we have begun to explore this part of the loop with our model. So far, results indicate that the emergence of the shape bias for solid items leads to an increase in the rate of shape-based word learning in particular (Sims et al., 2013). An open question is how the later emergence of the material bias for non-solid items influences the learning rate of different kinds of words. Further, once we have established predicted patterns of vocabulary growth in our model, we will test them in our longitudinal study of toddlers. Once completed, this work will have important implications for theories of word learning and the cognitive mechanisms that support this particular part of language development.

Our work focuses on modeling an entire trajectory of word learning and tracking the development of vocabulary and word learning biases at each step along the way. This method is powerful in that it allows us to look past children’s current learning and make predictions about language learning outcome. This direction has especially meaningful implications for work with different populations of children, such as those who are developmentally delayed. Thomas et al. (2009) have emphasized the importance of investigating trajectories of learning when studying developmental disorders. Through studies of children with Williams syndrome, Down syndrome, and autism spectrum disorder, these researchers argue that a trajectories approach is “descriptively powerful” for identifying developmental delays and factors that contribute to symptoms. As this work and others have done, we use the powerful approach of studying trajectories to focus on impaired development, particularly in the language development of late talkers. So far we have found evidence to support the idea that the vocabulary structures of late talkers as a group lead to the development of word learning biases that differ from those of early talkers and typically developing children. Next we must explore how these different trajectories unfold over time. That is, we want to go beyond modeling word learning biases at one arbitrary point in time, and instead to model the emergence of and interactions between attentional biases over developmental time among different populations. This work, and subsequent behavioral data analyses, will help to further elucidate when and how different populations of children diverge from one another along the trajectory of word learning.

All children are not the same, though they are often studied as a single population. In our investigations of late and early talkers, we attempt to target specific groups and identify useful differences in their learning styles. This research aims to separate children based on specific qualities, but what about going even further? Can we model children on an individual level? Work in other fields has also aimed for this goal. For example, Dell et al. (1997) fit a model of lexical networks to individual aphasic patient data. These authors adjusted connection weights and decay rate in each model to match performance levels of each patient. These individualized models were able to make predictions about performance on various speech processing and production tasks. Importantly these models allowed for predictions about patients on an individual level, which could be useful for diagnosis and intervention with specific patients. Similarly, Ziegler et al. (2008) developed a model of dyslexia which they fit to individuals by adjusting levels of noise. These simulations were able to both capture group level dyslexia profiles in the literature and account fairly well for individual reading patterns. As with Dell and colleagues’ work with aphasic patients, this kind of modeling work opens up the possibility of targeted intervention. In our own future work, we aim to pair this technique of modeling on the individual level with the study of trajectories of word learning. If we are able to model the trajectories of individual children as they learn new words and grow, we may be able to predict whether or not late talkers in particular will catch up with their peers or what specific intervention techniques could lead to this recovery. We want to utilize the information we have about a child, specifically the words that they know, at one point in time to predict how they will develop word learning biases and subsequently learn new words later in time. While we are confident in our current model’s ability to do this at a group level, we may need to further develop the model in order to explore these dynamics and make predictions at the level of individual children.

An additional benefit of modeling developmental trajectories is that it allows us to test predictions which would be difficult or impossible to test in children. Vitevitch and Storkel (2013) demonstrate this point in their model of phonological word learning. These authors implemented manipulations such as reducing cognitive resources and exposing the model to learning environments that might retard typical development. The effects of such manipulations on word learning would be unethical to implement in an experimental study of children, but can be investigated with modeling techniques. Similarly in our future work, we could implement unfavorable word learning environments or induce language impairment in our simulations. These models would allow us to more efficiently test intervention techniques before implementing them with real children. In this way, modeling trajectories of word learning with connectionist models could results in improved techniques of intervention and a proliferation of information on the causes of word learning deficits.

Looking at trajectories of word learning at both the group and individual level, and among different populations of children, will be vital for informing early language interventions. Our hope in investigating the dynamics of the developmental feedback loop in word learning is to pinpoint when and how the processes in this loop may be most receptive to intervention. Models of early, typical, and late talkers will help reveal when in developmental time and at what point in the developmental loop these populations differ in their word learning trajectories. Models of individual trajectories will help to demonstrate how different vocabulary structures lead to different word learning and attentional bias outcomes. Putting this information together, it may be possible to identify an ideal point in development at which to introduce certain types of words or to train certain types of biases in order to facilitate learning for children who would otherwise struggle. Our developmental model of word learning will be a crucial tool in guiding this work and in the creation of intervention strategies that we can test longitudinally with children.

## CONCLUSION

Connectionist models of language that aim to capture and explain developmental processes represent a powerful approach to language research. As reviewed here, such models have satisfied three modeling criteria; they have made good contact with data from real children, captured psychologically valid tasks, and accurately simulated characteristics of young children’s linguistic environments. Some of these models have also worked toward our fourth criteria for developmental models, temporality. They capture change in phenomena over time, providing novel insights into the dynamic processes that move children from one point in development to the next. This quality of temporality is important to strive for, particularly because of the potential to better understand and predict the course of development and eventual outcomes. In our model of word learning we have shown that there is continuous, dynamic interplay between the kinds of words learned and selective attentional biases to object features. Patterns of word learning and attentional biases may also provide signatures of learning differences among varied populations and possibly even between individual children. A connectionist modeling approach may help us better understand individual trajectories of language development and, more importantly, design personalized interventions for children who are struggling. Connectionist models of language development are an innovative tool for understanding, diagnosis, and intervention in children’s language learning.

## Conflict of Interest Statement

The authors declare that the research was conducted in the absence of any commercial or financial relationships that could be construed as a potential conflict of interest.

## Footnotes

1 For judgments of solidity, Colunga and Smith (2005) asked adult subjects to answer three questions about each word: (1) Do items named by the word change shape when pressed? (2) Do they return to their original shape after being pressed? (3) Do they take the shape of their container? Words were counted as solid if all questions were answered with “no” and as non-solid is all were answered with “yes.” Judgments of characteristic feature were originally gathered by Samuelson and Smith (1999) by asking adult subjects to “indicate for each word which perceptible properties were characteristic across instances of the named category” (p. 5–6)
